# 

**Published:** 1882-06

**Authors:** 


					﻿Whole Number,
CCXXXXI.
JUNE, 1882.
Number it,
Volume XXI,
THE
BUFFALO
Medical and Surgical Journal.
EDITORS :
THOS. I.OTHROP, M.
H. W. VAN PEVMA, M. I>.,
A. R. DAVIDSON, M. !>,,
LUCIEN HO WK, M. I*.,
HERMAN MYNTER, M. I>.
BTTFFA LO :
Published by the Medical Journal Association.
Read the Notice of this Journal among the Advertisements.
Entered at the Post-Office at Buffalo, N. Y., as Second-Class Mail Matter.
				

